# Exploring the Therapeutic Potential of Traditional Antimalarial and Antidengue Plants: A Mechanistic Perspective

**DOI:** 10.1155/2023/1860084

**Published:** 2023-10-28

**Authors:** Chinnaperumal Kamaraj, Chinnasamy Ragavendran, Pradisha Prem, Selvam Naveen Kumar, Amir Ali, Abeer Kazmi, Abd Ullah, Rajappan Chandra Satish Kumar, Safir Ullah Khan, Juan Pedro Luna-Arias, Zia-Ur-Rehman Mashwani, Govindhasamy Balasubramani, Saif Ur Rehman

**Affiliations:** ^1^Interdisciplinary Institute of Indian System of Medicine (IIISM), SRM Institute Science and Technology, Kattankulathur, Chennai 603203, Tamil Nadu, India; ^2^Department of Conservative Dentistry and Endodontics, Saveetha Dental College and Hospitals, Saveetha Institute of Medical and Technical Sciences (SIMATS), Chennai 600-77, India; ^3^Department of Biotechnology, Faculty of Science and Humanities, SRM Institute of Science and Technology (SRMIST), Kattankulatur, Chennai 603203, Tamil Nadu, India; ^4^Nanoscience and Nanotechnology Program Center for Research and Advanced Studies, National Polytechnic Institute, Mexico City, Mexico; ^5^Department of Cell Biology, Center for Research and Advanced Studies of the National Polytechnic Institute, Av. Instituto Politécnico Nacional 2508, Col. San Pedro Zacatenco C.P.07360, Mexico City, Mexico; ^6^Department of Botany, Pir Mehr Ali Shah Arid Agriculture University, Rawalpindi, Pakistan; ^7^The State Key Laboratory of Freshwater Ecology and Biotechnology, The Key Laboratory of Aquatic Biodiversity and Conservation of Chinese Academy of Sciences, Institute of Hydrobiology, Chinese Academy of Sciences, Wuhan 430072, Hubei, China; ^8^University of Chinese Academy of Sciences, Beijing 100049, China; ^9^Xinjiang Key Laboratory of Desert Plant Root Ecology and Vegetation Restoration, Xinjiang Institute of Ecology and Geography, Chinese Academy of Sciences, Urumqi, China; ^10^Department of Research and Innovation, Saveetha School of Engineering, Saveetha Institute of Medical and Technical Sciences, Chennai 603102, Tamil Nadu, India; ^11^Rahman Medical Laboratories, Kabul, Afghanistan

## Abstract

Malaria, a highly perilous infectious disease, impacted approximately 230 million individuals globally in 2019. Mosquitoes, vectors of over 10% of worldwide diseases, pose a significant public health menace. The pressing need for novel antimalarial drugs arises due to the imminent threat faced by nearly 40% of the global population and the escalating resistance of parasites to current treatments. This study comprehensively addresses prevalent parasitic and viral illnesses transmitted by mosquitoes, leading to the annual symptomatic infections of 400 million individuals, placing 100 million at constant risk of contracting these diseases. Extensive investigations underscore the pivotal role of traditional plants as rich sources for pioneering pharmaceuticals. The latter half of this century witnessed the ascent of bioactive compounds within traditional medicine, laying the foundation for modern therapeutic breakthroughs. Herbal medicine, notably influential in underdeveloped or developing nations, remains an essential healthcare resource. Traditional Indian medical systems such as Ayurveda, Siddha, and Unani, with a history of successful outcomes, highlight the potential of these methodologies. Current scrutiny of Indian medicinal herbs reveals their promise as cutting-edge drug reservoirs. The propensity of plant-derived compounds to interact with biological receptors positions them as prime candidates for drug development. Yet, a comprehensive perspective is crucial. While this study underscores the promise of plant-based compounds as therapeutic agents against malaria and dengue fever, acknowledging the intricate complexities of drug development and the challenges therein are imperative. The journey from traditional remedies to contemporary medical applications is multifaceted and warrants prudent consideration. This research aspires to offer invaluable insights into the management of malaria and dengue fever. By unveiling plant-based compounds with potential antimalarial and antiviral properties, this study aims to contribute to disease control. In pursuit of this goal, a thorough understanding of the mechanistic foundations of traditional antimalarial and antidengue plants opens doors to novel therapeutic avenues.

## 1. Introduction

Mosquitoes, those seemingly innocuous insects, hold the dubious distinction of being humanity's most formidable adversaries in the realm of public health. These tiny vectors, belonging to the Diptera Culicidae family, are the harbingers of a multitude of diseases that strike with devastating impact. Among their cargo are notorious maladies such as dengue, yellow fever, chikungunya, encephalitis, West Nile virus, and Zika virus. These infectious scourges have morphed into substantial threats, especially in equatorial regions, wreaking havoc not only on human lives but also on economies. Annually, more than 700,000 lives succumb to the relentless onslaught of vector-borne illnesses [1–3].

In the face of these mosquito-transmitted diseases, the absence of effective vaccines and specific antiviral medications leaves a void eagerly waiting to be filled. Herein lays the promise of natural compounds. With their anticipated minimal side effects, widespread availability, and potential efficacy, natural compounds emerge as beacons of hope, particularly for economically challenged nations [4]. Numerous studies have unveiled the antiviral prowess of various phytonutrients against pathogens such as the dengue virus (DENV) and chikungunya virus (CHIKV). Among these bioactive compounds are polysaccharides, alkaloids, terpenoids, terpenes, and flavonoids [4]. Interestingly, medicinal plants have showcased superior anti-DENV and CHIKV activity when compared to their synthetic counterparts [5]. The exploration of natural products is poised to become a preferred avenue for the development of novel therapeutics.

In in vitro tests involving THP-1 cells treated with papaya leaf extract (PLE), the antiviral activity was evident, leading to a notable reduction in viral load and an increase in the expression of type I interferon (IFN-*α*) [5]. Through in vivo studies conducted on DENV-infected mice, the treatment with papaya plant leaf extract demonstrated the modulation of genes related to regulating endothelial permeability in the liver [6, 7]. The identification of the active component responsible for the antidengue and antichikungunya effects will aid in the development of novel drugs. Studies have revealed that quercetin, a bioflavonoid found in papaya leaf extract, exhibits efficacy against DENV (Dengue virus) [6, 8].

As we grapple with the emergence of drug-resistant Plasmodium strains, the battle against malaria has taken on even greater urgency. Over the past two decades, research into alternative antimalarial strategies has gained momentum. Novel therapeutic targets for both treatment and prevention are becoming increasingly vital in our fight against this relentless adversary. Medicinal herbs, with their repository of natural products, have been foundational in traditional medicine for centuries. Indian systems of medicine, such as Ayurveda, Siddha, and Unani, boast a rich history of utilizing these natural products. These systems, which have evolved into well-organized and regulated practices, have significantly contributed to antimalarial medications, yielding compounds such as quinines, triterpenes, lapinone, quassinoids, and artemisinin [6]. The study of the effects of herbal plant products on humans provides valuable insights into novel molecular platforms, laying the groundwork for innovative antimalarial therapeutics [7].

*Aedes aegypti*, a prominent vector, thrives in and around polluted environments, often taking refuge amidst man-made refuse and exclusively seeking human blood [5]. In the realm of mosquito-borne diseases, dengue stands out prominently. An alarming 3.9 billion individuals across 129 countries are at risk of dengue infection, resulting in approximately 96 million symptomatic cases and an estimated 40,000 fatalities annually [4]. The primary culprits responsible for spreading the dengue virus are female mosquitoes, particularly *A. aegypti* and *A. albopictus*. These vectors, in addition to transmitting dengue, also serve as carriers for the Zika virus and are associated with other diseases such as chikungunya and yellow fever. Dengue fever casts its ominous shadow primarily over tropical regions, with varying degrees of risk dictated by factors such as rainfall, temperature, and unchecked urban expansion. The severity of the virus, manifested as dengue haemorrhagic fever (DHF), has made dengue a significant cause of suffering and death among both children and adults in several Asian and Latin American nations [6]. The dengue virus, classified into four serotypes (DEN-1, DEN-2, DEN-3, and DEN-4), presents a unique yet closely related quartet [7, 8].

Malaria, a potentially life-threatening malady, is inflicted by an array of parasites, including *Plasmodium vivax*, *Plasmodium falciparum*, *Plasmodium malariae*, and *Plasmodium ovale*. Each year, this disease claims the lives of over 400,000 individuals and afflicts an estimated 219 million people worldwide. Tragically, the majority of fatalities involve children under the age of five [4]. The transmission of malaria is orchestrated by the Anopheles mosquito. In India, two common culprits for human malaria are *Plasmodium vivax* and *Plasmodium falciparum* [8]. Within the human host, the parasite embarks on a complex journey, undergoing a series of transformations throughout its intricate life cycle, which encompasses pre-erythrocytic and erythrocytic schizogony stages. Among the various strains of malaria, *P. falciparum* stands out as the most ominous, capable of causing severe infections that, if left untreated, can escalate into the deadliest form of the disease [9].

Against this backdrop, this study aims to delve into the rich repository of medicinal plants used in the treatment of malaria and dengue fever in humans. The exploration of plant-based compounds with antimalarial and antiviral properties holds immense promise, paving the way for novel therapeutics in the fight against these formidable diseases.

## 2. Global Burden and Epidemiology of Dengue

In recent decades, the global prevalence of dengue fever has surged, with alarming statistics painting a grim picture. Current estimates suggest that approximately 390 million dengue infections occur each year, spanning a wide range from 284 to 528 million, with around 96 million cases exhibiting varying degrees of clinical symptoms [4, 7]. Shockingly, more than 3 billion individuals now find themselves under the looming threat of dengue virus infection [8].

**Figure 1:**
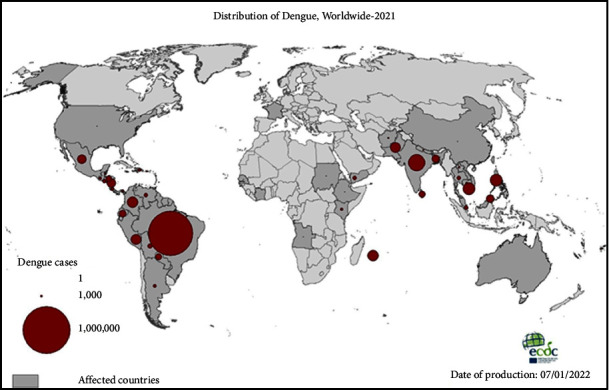
Dengue distribution in 2021, the five countries reporting the most cases were Brazil, India, Vietnam, the Philippines, and Colombia. (source: ECDPC, 2021).

It is worth noting that before 1970, dengue outbreaks were confined to just nine countries. However, since 2010, this insidious disease has spread its tendrils to over a hundred countries within the WHO region. The reported cases have ballooned, escalating from 2.2 million in 2010 to a staggering estimated 3.2 million in 2015 [6] (see Figure 1 for the average number of dengue cases reported to ECDPC globally in 2021). In 2015 alone, Delhi, India, grappled with over 15,000 cases of dengue fever, and in 2017, the National Vector Borne Disease Control Program (NVBDCP) recorded a staggering total of 1,096,76 dengue cases, accompanied by 187 fatalities [9, 10].

This relentless increase in dengue's global prevalence has captured the attention of the world. Dengue fever has firmly established itself in tropical and subtropical regions, with a particularly high incidence in urban and semiurban areas worldwide [11, 12]. As defined by the WHO in 2016, dengue fever is a febrile illness that can afflict both infants and adults. The onset of symptoms typically occurs between 3 and 14 days following the bite of an infected mosquito. While dengue is not transmitted from person to person, its symptoms can range from a mild fever to a perilously high temperature, often accompanied by severe headaches, eye discomfort, muscle and joint pain, and a distinctive rash. Presently, there is neither a dengue vaccine nor any specific treatment recommended by the WHO. However, under medical supervision, paracetamol may be used to alleviate fever in individuals with dengue fever [6].

## 3. Global Burden and Epidemiology of Malaria

The shadow of malaria continues to loom large over public health worldwide, as underscored by the World Health Organization's World Malaria Report for the year 2022. In 2021, malarial infections remained endemic in a staggering 84 countries, painting a vivid picture of the persistent threat [13]. What's particularly concerning is the intersection of the COVID-19 pandemic with the malaria burden. Between 2019 and 2021, the world witnessed an additional 13.4 million cases of malaria and a heartbreaking 63,000 more fatalities, a grim testament to the challenges posed by overlapping health crises.

**Figure 2:**
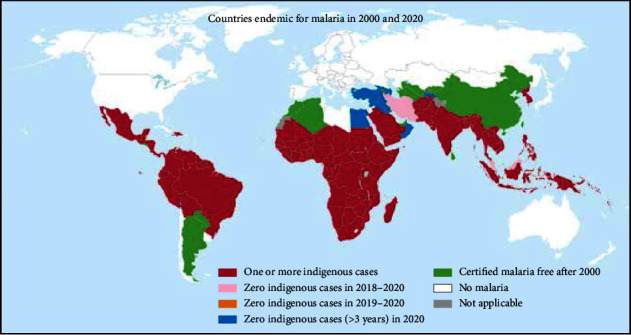
Malaria endemic, 2000–2020 (source: WHO, Malaria Report, 2022).

While there was a glimmer of hope in the earlier part of the past decade, with a steady decrease in the global burden of malaria from 2010 to 2015, the subsequent years have brought sobering setbacks. The number of reported malaria cases globally surged, rising from 232 million in 2019 to 245 million in 2020, and further to 247 million in 2021. The toll on lives has also been profound, with fatalities climbing from 568,000 in 2019 to 625,000 in 2020 before receding slightly to 619,000 in 2021 [13] (see Figure 2 for a visual representation of the global endemicity of malaria in 2000–2020).

In 2015, malaria exacted a staggering toll of approximately 429,000 lives, with a heavy concentration in Africa (92%), South-East Asia (6%), and the Eastern Mediterranean (3%) [4]. Of particular concern is the toll it takes on our youngest population. In the same year, an estimated 303,000 children under the age of five succumbed to this disease, with a heart-wrenching 292,000 of these tragedies unfolding in Africa. Indeed, malaria remains the chief cause of death among children under five, snatching away a child's life every two minutes [4].

## 4. Malaria Distribution in India

**Figure 3:**
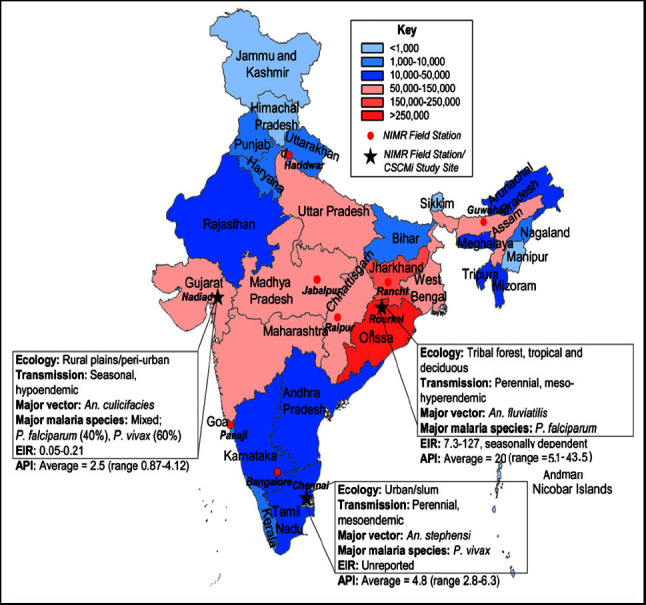
Malaria endemicity in India. State boundaries are color-coded according to total malaria endemicity. NIMR; National Institute of Malaria Research Field Stations are indicated as red dots; CSCMi; Center for the Study of Complex Malaria in India; EIR: entomological inoculation rate; API, annual parasite incidence [15].

The specter of malaria continues to cast a long shadow over public health, with numbers that demand attention. Back in 1990, approximately 75 million Indians stood at high risk of contracting malaria, while another 240 million lived under moderate risk, and a staggering 500 million had a relatively low risk [14]. This formidable challenge is further accentuated by the fact that nearly two-thirds of individuals living in Southeast Asia, a region encompassing India, are exposed to the threat of malaria [14].  Figure 3 illustrates the prevalence of malaria in specific regions of India, namely, the Central and Eastern regions, which include the states of Orissa, West Bengal, and Jharkhand. In addition, the Central states of Chhattisgarh and Madhya Pradesh, along with the Western states of Gujarat, Karnataka, and Rajasthan, also show significant malaria cases. Among these regions, Orissa reported the highest number of malaria-related deaths [16]. Certain regions of India experience a favorable combination of average temperatures ranging from 15 to 30°C, consistent rainfall, and precipitation-inducing conditions throughout the year, creating conducive environments for the abundance of malaria cases.

## 5. The Resurgence and Globalization of Indian Traditional Medicine

The past few years have witnessed an extraordinary surge in the utilization of traditional and complementary medicine on a global scale. These therapies have not only found their place in the hearts of communities but have also made profound inroads into modern healthcare systems. The statistics paint a vivid picture: nearly 80% of Africa's population now relies on traditional medicine as their primary healthcare [17]. In China, a country with a rich tradition of herbal remedies, an estimated 30 to 50% of all medicinal use stems from traditional herbal medicines. In West African nations like Ghana, Mali, Nigeria, and Zambia, traditional herbal medicines are the go-to treatment for malaria-induced high fevers, trusted by almost 60% of the population [17].

The trend is not limited to specific regions; it is a global phenomenon. Approximately 48% of individuals in Australia, 70% in Canada, 80% in Germany, 42% in the United States, 39% in Belgium, and a striking 76% in France have reported using traditional or complementary medicine at least once [18, 19]. In the cosmopolitan centers of London, San Francisco, and South Africa, traditional and complementary medicine have found a solid footing, with more than 75% of individuals living with HIV/AIDS turning to these therapies for holistic care. Even in the bustling urban landscapes of Malaysia, the expenditure on traditional medicine surpasses that on pharmaceuticals.

**Figure 4:**
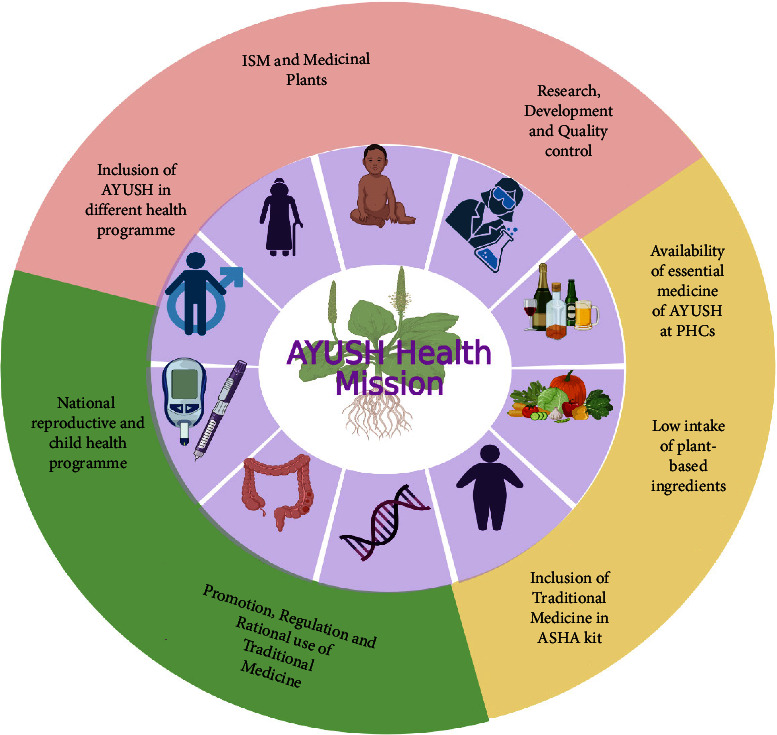
Integrating traditional medicine into mainstream healthcare services in India: Ayush Health Mission's strategic approach.

But what lies at the heart of this resurgence of interest in traditional medicine? It is not merely a return to ancient practices; it is a testament to the growing recognition of the holistic benefits these therapies offer. The increasing use of traditional medicine has transformed healthcare systems, offering a holistic approach that addresses not only physical ailments but also mental and spiritual well-being [17–19]. In an era where healthcare is often synonymous with pharmaceuticals, traditional herbal medications are emerging as vital components, not just for individual well-being but also for the broader economy [17–19].  Figure 4 presents an example of how traditional herbal therapy has been integrated into clinical practice.

## 6. Indian System of Medicine (ISM), Medicinal Plants of India, and Economy

The Indian system of medicine (ISM) boasts an impressive arsenal of approximately 25,000 effective plant-based formulations. These remedies have long been the trusted choice of rural and ethnic communities in India, and their popularity is steadily growing among the general population. The demand for these medicinal plant materials is substantial, with an estimated yearly requirement exceeding 2,000 tonnes. In this vast landscape, over 1,500 herbal products are available as dietary supplements or traditional ethnic treatments, and many are marketed commercially [5].

Amid this flourishing industry, about 960 species of medicinal plants actively participate in commerce, with 178 of these species having an annual consumption exceeding 100 metric tonnes. The economic significance of this sector is profound, with approximately INR 80–90 billion traded within the AYUSH (Ayurveda, Yoga and Naturopathy, Unani, Siddha, and Homeopathy) sector domestically and an additional INR 110 billion exported from India in the form of medicinal plants and allied items. Although there was a slight decrease in exports in the financial year 2013-2014 compared to the previous year, INR 24,741.2 crores worth of goods were exported in 2012-2013. Remarkably, the AYUSH items constituted 0.36% of India's overall commerce in 2013-2014. Against the backdrop of this expansive growth, it is noteworthy that the global herbal trade is projected to reach an astonishing $7 trillion in sales worldwide by the year 2050 [20–22].

## 7. Indian Medicinal Plants and Drug Discovery

**Figure 5:**
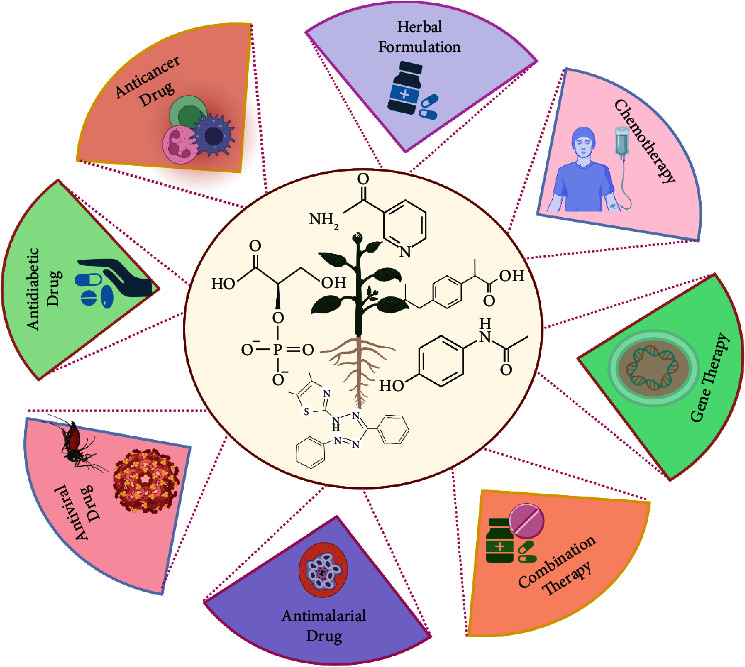
The opportunity for drug discovery from Indian traditional medicinal plants.

It is becoming more popular to conduct drug discovery studies using botanicals traditionally employed in various Indian healing traditions. As a result of employing natural substances as a starting point, several new medications have been found. Such a study relied heavily on investigations both in India and overseas. *Asparagus adscends* has many medicinally significant compounds that date back to ancient times, including vasicine and vasicinone, derived from *Adhatoda vasica*, Homoharringtonine, obtained from *Cephalotaxus*, morphine, and Camptothecin, discovered in *Camptotheca acuminate*, Bacosoids, extracted from *Bacopa monnieri*, Codeine, derived from *Papaver somniferum*, Tylophorine, found in *Tylophora indica*, Sarsasapogenin, Asparanin A, and Asparanin B, and isolated from *Asparagus adscendens*. Likely, some of the findings [23–25] are summarised. Catechin, extracted from *Acacia catechu*, Trigonelline is found in *Trigonella foenumgraecum*, Shatavarin, obtained from *Asparagus racemosus*, Glycyrrhizin, extracted from *Glycyrrhiza glabra*, Atropine, found in *Atropa belladonna*, cocaine, derived from *Erythroxylum coca*, Tinosporic acid, discovered in *Tinospora cordifolia*, Sophoradin, obtained from *Sophora subprostrata*, Aloin, derived from Aloe vera, Quinine, derived from *Cinchona spp*., Protodioscin, discovered in *Tribulu sterrestris*, Aegelin, and marmelosin, isolated from *Aeglemarmelos*, Withanolides, obtained from *Withania somnifera*, Plumbagin, found in *Plumbago indica*, Arjunolic acid, derived from *Terminalia arjuna*, Jatamansone, found in *Nardostachys jatamansi*, Quassinoids, obtained from *Ailanthus spp*., Asiaticoside, found in *Centella asiatica*, Boeravinones, discovered in *Boerhavia diffusa*, Curcumin, extracted from *Curcuma longa*, Forskolin, extracted from *Coleus forskohlii*, Paclitaxel, extracted from *Taxus baccata* and *Taxus brevifolia*, Gingerols, found in *Zingiber officinale*, Emetine, derived from *Cephaelis ipecacuanha*, Digoxin and digitoxin isolated from *Digitalis lantana*, Dysobinin, obtained from *Dysoxylum binectariferum,* Psoralen, extracted from *Psoralea corylifolia*, Nimbidin, found in *Azadirachta indica*, Berberine, derived from *Berberis aristate*, Allicin, obtained from *Allium sativum*, Podophyllin, derived from *Podophyllum emodi*, Pilocarpine, derived from *Pilocarpus jaborandi*, Vinblastine and vincristine, extracted from *Catharanthus roseus*, and Diosgenin, found in *Dioscorea spp*. and many more [24, 25]. More and more medications and formulations from the public and commercial sectors in India have been tested in clinical trials in recent years. India provides a huge selection of medicinal plants and a lengthy and well-documented traditional medical system, making it an ideal location for novel drug development. Throughout the Indian system of medicine, there are several avenues for drug development (Figure 5).

## 8. Dengue-Treating Plants from Folklore and Herbal Medicine

According to World Health Organization (WHO) information from December 2008, certain African and Asian countries heavily rely on the use of conventional treatment as their main healthcare system due to its economic limitations and geographic restrictions [14]. Natural materials have emerged as the predominant source of test material in the development of antiviral medications, following conventional medical practices [26]. Traditional medicines are founded on indigenous cultural beliefs and practices and employ knowledge, experience, and traditions to keep people well and cure, prevent, and diagnose physical and mental conditions [14]. The World Health Organization has demonstrated that traditional medicinal herbs possess antiviral properties [27, 28]. Dengue may be treated by several different species, some of which have yet to be formally researched (as depicted in Table 1).

The folk remedy *Euphorbia hirta* and *Uvaria chamae* are used to treat dengue disease in the Philippines by rural residents [42]. Practitioners of traditional medicine think that *E. hirta* leaves may cure the viral disease and prevent the fever from advancing into severe stages, but no scientific investigations have shown its efficacy [43]. In certain cases, *E. hirta* and papaya leaf extract are combined to make *E. hirta,* which is employed as an antibiotic for treating fever. In addition to killing the germs that produce fever, *E. Hirta* and Aloe vera extracts help stop bleeding. *Psidium guava* leaves have also been reported to raise platelets, thereby reducing the risk of blood clots [43, 44]. Guava leaves contain quercetin, which inhibits the virus's ability to produce enzyme mRNA. This plant's leaves are rich in andrographolide, a labdane diterpenoid that has been shown to exhibit effectiveness against the chikungunya virus (CHIKV) and dengue virus serotypes 2 and 4, as well as other viruses [29, 45]. According to a literature search, a total of twenty-two plants from around India were discovered to be effective against dengue fever. Further clinical trials are necessary to validate the safety, effectiveness, and method of action of additional plants that have been reported [46, 47]. During the validation research, these plants may undergo testing either individually or in various combinations to gain a deeper understanding of their synergistic effects in terms of both efficacy and safety (Table 2).

## 9. Antimalarial Properties of Natural Items

To comprehend the origins of antimalarial chemotherapy, it is essential to delve into the historical background of herbal medicines. Antimalarial chemotherapy was first made possible by the discovery of Quinine, a natural compound extracted from the bark of the Cinchona tree and subsequently refined in 1820 [69]. To manufacture quinine, researchers discovered methylene blue, which led to the birth of the dye business. The development of chloroquine, amodiaquine, and mefloquine, which have been the foundation of malaria therapy for the past century, can be attributed to this advancement [70]. *Naphthoquinone lapichol*, a natural substance, was also shown to be the active component in tree rind used to cure malaria [71]. It was because of this finding that lapinone was chosen as a starting point for the development of atovaquone, a component of Malarone® and a staple of malaria prophylaxis for travelers to this day [72]. When the sweet wormwood, *Artemisia annua*, was originally identified in 1971, it contained the compound artemisinin. These long-acting, totally synthetic compounds derived from a unique endoperoxide group are now under clinical development [73] and may provide the foundation for future medicines to combat artemisinin-resistant malaria.

More than a few nations in Africa, the Americas, and Asia have long relied on botanicals to cure malaria [74]. There is, however, a dearth of scientific evidence supporting the use of these herbs as medicine. Because of their significant antiplasmodial activity, medicinal plant extracts may be used to identify novel lead structures for drug development by bioassay-guided extraction [75]. India, possessing a wide range of medicinal plants, is recognized as one of the twelve mega-biodiversity nations worldwide [76]. To the best of our understanding, pharmacological tests have been used in several earlier studies to substantiate the global flora. As indicated in Table 3, certain medicinal plants' components have antiplasmodial activity. *Phyllanthus emblica*, *Phyllanthus acidus*, and *Leucas aspera* leaf ethyl acetate and methanolic extracts showed potential antiplasmodial action against CQ-sensitive (3D7) and CQ-resistant (D2) strains of *P. falciparum*, according to the research [95]. The process of isolating and purifying antimalarial compounds involved bioassay-guided chromatographic fractionation from the leaves of *Murraya koenigii* and showed potential activity against *P. falciparum* (3D7) and *in vivo* mice infected with *P. berghei* (NK65) strains [96]. Extractions of *Toddalia asiatica*'*s* root leaves, bark, and fruits in ethyl and methanol demonstrated effective antimalarial activity against *P. falciparum*CQ-sensitive (D6) and CQ-resistant (W2) strains [97]. *Aegle marmelos*, *Lantana camara*, *Leucas aspera*, *Momordica charantia*, *Phyllanthus amarus,* and *Piper nigrum* extract showed the highest activity against CQ-sensitive 3D7 and CQ-resistant INDO strains of *P. falciparum* [98]. It was shown that urospermal A-15-O-acetate, which was isolated from the leaf of *Dicoma tomentosa*, has good in vitro antiplasmodial activity against *P. falciparum* strains CQ-sensitive and CQ-resistant (3D7 and W2), respectively. Good antiplasmodial activity against the CQ-resistant (2/K1) strain of *P. falciparum* was found in the methanol extracts of the leaves and stems of *Chrozophora oblongifolia, Ficus ingens, Plectranthus barbatus,* and *Lavandula dentate* [99].

## 10. Utilizing Medicinal Herbs for Nanoparticle Synthesis

**Figure 6:**
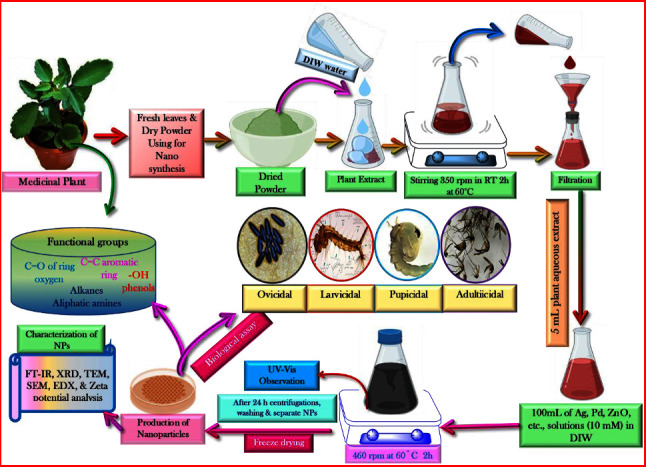
Synthesis of nanoparticles using medicinal plants.

**Figure 7:**
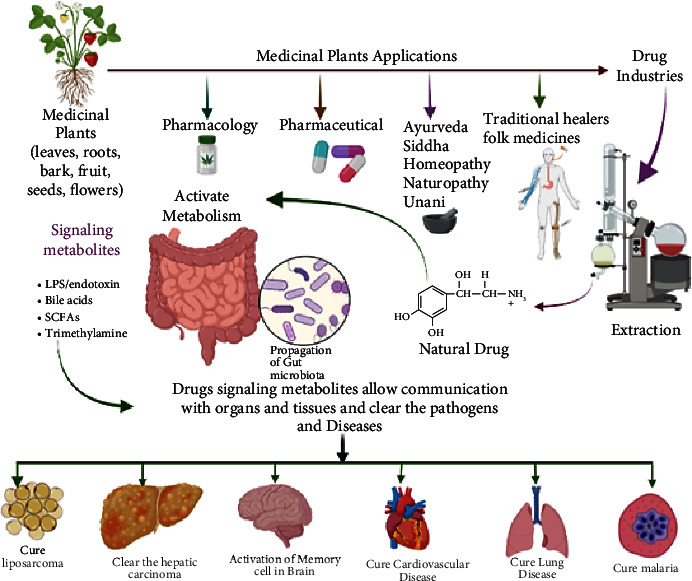
The diverse applications of medicinal plants are attributed to the presence of profoundly bioactive secondary metabolites.

*Anopheles stephensi* mosquitoes transmit *P. falciparum*, which causes chikungunya, dengue, malaria, yellow fever, Zika fever, Japanese encephalitis, and lymphatic filariasis among other awful illnesses, to humans [100]. Many areas, including industrial, pharmaceutical, and medical, rely on research into the production of bio-metal nanoparticles [101]. This encompasses both diagnostics and therapies, exhibiting a wide array of in vitro properties such as antibacterial, cytotoxic, and antibiofilm efficacy, as well as impacting inflammatory and coagulation responses. In addition, they target drug delivery, demonstrate antiplasmodial activity, and exhibit activity against medically significant pathogens and formidable vectors such as mosquitoes [102] (Figures 6 and 7).  Table 4 shows past studies on the synthesis and characterization of nanoparticles generated utilizing medicinal herbs. *Artimisia nilagirica* leaf filtrate-based biological silver nanoparticle manufacturing might be a viable green larvicidal option for the control of mosquitoes throughout the development phases of the malaria vector *A. stephensi* and the dengue vector *Aedes anaxyrus* [103]. *A. stephensi* larvae and pupae were killed by both the *Ulva lactuca* extract and the green-synthesized AgNP, both of which were examined [115, 116]. All of these plants were submitted to green silver nanoparticle production and tested for their ability to kill *A. stephensi* mosquitoes, which cause malaria, as well as their larvicidal efficacy against *A. stephensi* larvae [105]. *Suaeda maritima*-synthesized AgNP demonstrated larvicidal and pupicidal effects against the dengue vector *Aedes aegypti* [106]. *Sargassum wightii* extract and ZnO NP exhibited larvicidal and pupicidal toxicity against the malaria vector *Aedes stephensi*. As of 2018, Nalini et al. [104] used *Cleistanthus collinus* and *Strychnos nuxvomica* plant leaf extracts which were used to create metallic silver nanohybrids (AgNPs) against chikungunya, dengue, and Zika vectors. According to Murugan et al. [108], the antimalaria and antidengue vector larvicidal functions of silver nanoparticles (AgNPs) utilize *Belosynapsis kewensis* leaf extracts. Bhuvaneswari et al. [110] found that silver nanoparticles (AgNPs) derived from Chrysanthemum are effective against the mosquito that transmits dengue [111, 117]. Larvicidal effectiveness against malaria, dengue, and filariasis vectored by *Culex quinquefaciatus* was studied via the production of silver nanoparticles from aqueous leaf extracts of *Leucas aspera* and *Hyptis suaveolens*, respectively, and their ability to reduce *C. quinquefaciatus* to silver nanoparticles [111]. Silver nanoparticles produced from *Annona squamosa* contain isoamyl acetate, which has larvicidal properties against mosquitoes that transmit dengue fever (Aegypti) and filariasis (*C. quinquefaciatus*). [112, 118]. Environmentally friendly nanopesticides like *Artemisia vulgaris* might be used to make gold nanoparticles [119, 120]. Against Zika virus mosquito vector *A. aegypti*, an old Indian medicinal plant called *Pedalium murex* was used to produce silver nanoparticles [115, 121]. In this figure, medicinal herbs are used to synthesize nanoparticles.

## 11. Bridging Nature and Science: A Promising Future for Infectious Disease Treatment

Natural products continue to serve as an abundant and untapped reservoir of chemical diversity, offering potential as a primary origin for the emergence of new therapeutic agents to address various health conditions, including infectious disorders. New approaches to combating dengue and malaria might result from accepting and validating traditional medicinal methods and hunting for plant-derived medications. So, the hunt for innovative and, if possible, low-cost medications must continue. Dengue and malaria parasites, which are becoming harder to treat because of resistance to currently available treatments, must be eradicated via the development of safe and economical drugs. The customary use of many plants and their preparations in various areas of India for dengue and malaria has been documented. Indian researchers employed a comprehensive approach, including clinical trials and in vivo and in vitro studies, to scientifically validate emerging herbal alternatives to examine their effectiveness against dengue viruses and malarial parasites. Dengue and malaria-fighting plants have received very few such scientific validations in India. Only a few herbs and extracts have been professionally researched and shown to be effective. For antidengue and antimalarial effectiveness and safety to be confirmed, more local herbal formulations need to be sought after, documented, and scientifically validated. This will help us better understand how these formulations work. If the appropriate steps of product creation, validation, and value addition are followed, herbal formulations already used by communities and healers could potentially offer alternative and supplementary therapies.

## Figures and Tables

**Table 1:** Medicinal plants are reported globally for their antidengue activity.

Plant species	Part(s) used	Extracts tested	References
*Andrographis paniculata*	Leaves	Andrographolide	[29]
*Alternanthera philoxeroides*	Whole plants	Petroleum ether, ethyl ether, ethyl acetate, and coumarin extract	[30]
*Cladosiphon okamuranus*	Whole plants	Ethanol extract	[31]
*Cladogynos orientalis*	Whole plants	Ethanol extract	[32]
*Leucaena leucocephala*	Seeds	Aqueous extract	[33, 34]
*Tephrosia crassifolia*	Leave and flowers	Flavonoid extract	[35]
*Tephrosia madrensis*	Leaves and flowers	Flavonoid extract	[35]
*Tephrosia viridiflora*	Leave and flowers	Flavonoid extract	[35]
*Quercus lusitanica*	Seeds	Methanol extract	[36]
*Cryptonemia crenulata*	Whole plants	Polysaccharide extract	[37]
*Gymnogongrus griffithsiae*	Whole plants	Polysaccharide extract	[37]
*Piper retrofractum*	Whole plants	Dichloromethane and ethanol extract	[32, 38]
*Rhizophora apiculata*	Whole plants	Ethanol extract	[32]
*Meristiellagelidium*	Whole plants	Polysaccharide extract	[39]
*Lippia alba*	Whole plants	Essential oils	[26, 40]
*Lippia citriodora*	Whole plants	Essential oils	[40]
*Zostera marina*	Whole plants	p-Sulfoxy-cinnamic acid and zosteric acid	[41]
*Uvariachamae p. Beauv.*	Dried leaves and fresh fruits	Methanol extract	[42]
*Aloe vera*	Leaves	Ethyl acetate and methanol	[43]

**Table 2:** Medicinal plants were reported to India for their activity against the dengue virus.

Plant species	Part/extract used	Details of study/report	References
*Azadirachta indica*	Leaves extract	In vitro and in vivo studies done against dengue virus type-2 showed positive effect in the reduction of virus	[48]
	Leaves extract	Used by tribal and traditional healers in various districts of Bihar	[49]

*Hippophae rhamnoides*	Leaves extract	In vitro assay against dengue virus type-2; the extract has a significant antidengue activity	[50]

*Carica papaya*	Leaves extract	Increase in platelet count in dengue patients and management of the disease	[51–56]
		Relevant uses in Ayurveda, local people of the North Eastern plain zone of India traditional healers, and local people of Goa, Madhya Pradesh, Uttar Pradesh, and Odisha	
		Clinical trials on patients indicate an increase in platelets due to the administration of the extract	

*Solanum virginianum*	Leaves decoction along with pepper and ginger	Used by the Santhal community in West Bengal	[57]

*Tinospora cordifolia*	Stem decoction and leaves	Used by the Gujjar community in Trikuta hills, Jammu and Kashmir, and tribal and local people of Bijnor in UP and Chhattisgarh	[58–60]

*Ocimum sanctum*	Leaves	Reported for its use as leave decoction	[61, 62]

*Andrographis paniculata*	Leaf, bark, and whole plant	Used by traditional healers in various districts of Bihar. Used by traditional healers in South India and in formulations	[63]
*Alternanthera sessilis*			
*Achyranthus aspera*			
*Calotropis procera*			
*Solanum xanthocarpum*			
*Plectranthus vettiveroides*			

*Brassica juncea*	Whole plant	Used by traditional healers of Nimari communities of Madhya Pradesh	[64]

*Adhatoda vasica*	Leaves extract	Reported for its use in formulation	[65]

*Euphorbia hirta*	Whole plant, root, leaves, and powdered seed	Used by traditional healers and communities of Uttar Pradesh, reported for its use as tonic and decoction or infusion, the extract was tested in in vitro and in vivo conditions and found effective against all four serotypes	[66]
*Cassia fistula*			
*Swertia chirata*			
*Datura metel*			
*Coriandrum sativum*			
*Peganum harmala*			
*Abutilon indicum*			
*Cissampelos pareira*			

*Andrographis paniculate* Nees	Whole plant	Treated to chikungunya virus and dengue virus	[67]
*Curcuma caesiarhizome extracts*	Whole plant	Antioxidant and antibacterial activity	[68]

*Leucas cephalotes (Roth.)*	Whole plant	Treated to malaria, dengue, and other types of fever	[69]

**Table 3:** Antiplasmodial activity of isolated compounds from medicinal plant extracts against *P. falciparum*.

Constituents	Botanical name	Antimalarial activity IC_50_ in *μ*g/ml (^a^) or *μ*M (^b^)	Compound structure	References
Anonaine (**7**)	*Goniothalamus australis*	7.0 *μ*M (3D7)	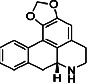	[77]

Stephanine (**1**) and pseudopalmatine (**8**)	*Stephania rotunda*	1.3 and 1.0 *μ*g/mL (W2)	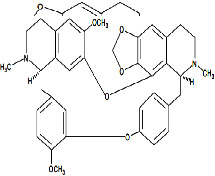	[78]

Catechin-[5,6-e]-4b-(3,4-dihydroxyphenyl)dihydro-2(3H)-pyranone (**3**)	*Flacourtia indica*	1.1 and > 5 *μ*g/mL (3D7 and K1)	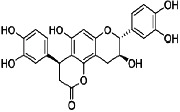	[79]

Ergosta-4,6,8,22-tetraene-3-one (**4**)	*Cornus florida*	61.0 *μ*M (D10)	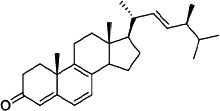	[80]

Methylenebissantin (**1)**	*Dodonaea viscosa*	91.13 *μ*M	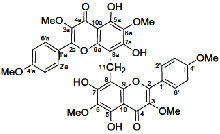	[81]

Sesquiterpene lactone de hydrobrachylaenolide	*Dicoma anomala*	1.865 and 4.095 *μ*M (D10 and K1)	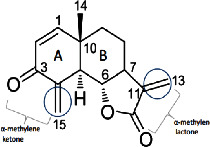	[82]

2,3,6-Trihydroxy benzoic acid and 2,3,6-trihydroxy methyl benzoate	*Sorindeia juglandifolia*	16.5 and 13.0 *μ*M (W2) ED_50_ 44.9 and 42.2 mg/kg (^B^)	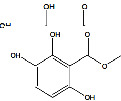	[83]

Strychnochrysine	*Strychnos nux-vomica*	4.9 and 6.0 *μ*M (3D7 and W2)	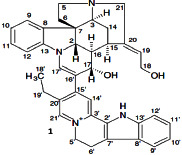	[84]

11*α*-Hydroxymuzigadiolide	*Warburgia ugandensis*	6.40^b^ (3D7)	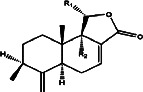	[85]

Dimethylisoborreverine	*Flindersia amboinensis*	0.22^a^ (3D7),	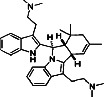	[86]
		0.02^a^ (FCR3),		
		0.81^a^ (HB3),		
		0.06^a^ (K1)		

Otogirin	*Hypericum erectum*	>50^b^ (D10)	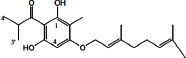	[87]

Otogirone	*H. erectum*	5.6^b^ (D10)	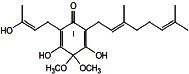	[87]

Erectquione A	*H. erectum*	11.2^b^ (D10)	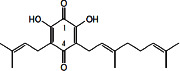	[87]

Erectquione B	*H. erectum*	7.2^b^ (D10)	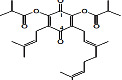	[87]

Erectquione C	*H. erectum*	13.2^b^ (D10)	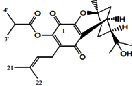	[87]

(+)-4′-Decanoyl-cis-khellactone	*Agelica purpuraefolia*	1.5^b^ (D10)	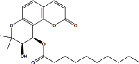	[88]

(+)-3′-Decanoyl-cis- khellactone	*A. purpuraefolia*	2.4^b^ (D10)	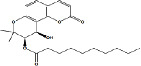	[88]

(+)-3-Acetylaltholactone	*Goniothalamus laoticus*	2.6^a^ (K1)	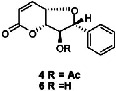	[89]

(−)-Nordicentrine	*G. laoticus*	0.3^a^ (K1)	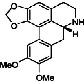	[89]

Dalparvone	*Dalbergia parviflora*	8.19^a^ (K1)	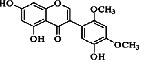	[90]

2-Isopropenyl-6-acetyl-8-methoxy-1, 3-benzodioxin-4-one	*Carpesium divaricatum*	2.3 ± 0.3^b^ (D10)	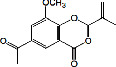	[91]

Oleifolisides A: *R*_1_ = *α*-arabinose, *R*_2_ = *β*-xylose and Oleifolisides B: *R*_1_ = *β*-arabinose, *R*_2_ = *β*-xylose	*Dendropanax morbifera*	24.4^b^ (D10)	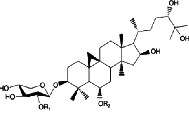	[92]
		6.2^b^ (D10)		

Dendropanoxide	*D. morbifera*	5.3^b^ (D10)	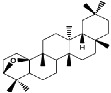	[92]

Atisinium chloride	*Aconitum chryseum*	4.0^b^ (TM4)	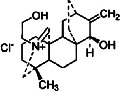	[93]
		3.6^b^ (K1)		

Norushinsunine	*Liriodendron tulipifera*	29.6^a^ (D10)	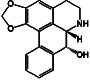	[94]

Norglaucine	*L. tulipifera*	22.0^a^ (D10)	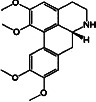	[94]

Oxoglaucine	*L. tulipifera*	9.1^a^ (D10)	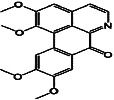	[94]

Peroxyferolide	*L. tulipifera*	6.2^a^ (D10)	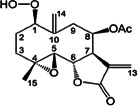	[94]

Lipiferolide	*L. tulipifera*	1.8^a^ (D10)	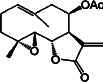	[94]

*Note*. Chloroquine, sensitive strains, 3D7, D10, FSG, and TM4; chloroquine, resistant strains, K1 and FCR3; pyrimethamine, resistant strains, K1 and HB3.

**Table 4:** Syntheses of nanoparticles using different medicinal plant extracts.

Plant species	Part(s) used	NPs used	Tested organisms	References
*Artimisia nilagirica*	Leaf	Silver nanoparticle	Malaria and dengue vectors (*Anopheles stephensi* and *Aedes aegypti)*	[103]
*Ulva lactuca*	Whole plant	Silver nanoparticle	Malaria vector (*Anopheles stephensi)*	[104]
*Phyllanthus amarus*, *Annona squamosa*, *Coccinia grandis*, and *Eclipta prostrate*	Whole plant	Silver nanoparticle	Malaria vector (*Anopheles stephensi)*	[105]
*Suaeda maritime*	Whole plant	Silver nanoparticle	Dengue vector *Aedes aegypti*	[106]
*Sargassum wightii*	Whole plant	Zinc oxide nanoparticle	Malaria vector (*Anopheles stephensi)*	[107]
C*leistanthus collinus* and *Strychnos nuxvomica*	Leaf extract	Silver nanoparticle	Dengue, chikungunya, and Zika vectors	[108]
*Belosynapsis kewensis*	Leaf extract	Silver nanoparticle	Malaria and dengue vectors (*Anopheles stephensi* and *Aedes aegypti)*	[109]
*Chrysanthemum*	Whole plant	Silver nanoparticle	Dengue vector (*Aedes aegypti*)	[110]
*Leucas aspera* and *Hyptis suaveolens*	Leaf extract	Silver nanoparticles	Malaria, dengue, and filariasis vectors (*Anopheles stephensi Aedes aegypti,* and *Culex quinquefaciatus)*	[111]
*Annona squamosa*	Whole plant	Silver nanoparticles	Dengue and filariasis vectors (*Aedes aegypti* and *Culex quinquefaciatus)*	[112]
*Artemisia vulgaris*	Whole plant	Gold nanoparticles	Dengue vector (*Aedes aegypti*)	[113]
*Pedalium murex*	Whole plant	Silver nanoparticles	Zika virus vector (*Aedes aegypti*)	[114]

## Data Availability

All the data used to support the findings of the study are available within the article.
